# In Vitro and In Vivo Studies of Hydrogenated Titanium Dioxide Nanotubes with Superhydrophilic Surfaces during Early Osseointegration

**DOI:** 10.3390/cells11213417

**Published:** 2022-10-28

**Authors:** Caiyun Wang, Shang Gao, Ran Lu, Xin Wang, Su Chen

**Affiliations:** 1 Laboratory of Biomaterials and Biomechanics, Beijing Stomatological Hospital, Capital Medical University, Beijing 100050, China; 2 Stomatology Department, Peking University Third Hospital, Beijing 100191, China

**Keywords:** bone mesenchymal stem cells, osteogenic differentiation, osseointegration, titanium implant, nanotopography, superhydrophilic nanotubes

## Abstract

Titanium-based implants are often utilized in oral implantology and craniofacial reconstructions. However, the biological inertness of machined titanium commonly results in unsatisfactory osseointegration. To improve the osseointegration properties, we modified the titanium implants with nanotubular/superhydrophilic surfaces through anodic oxidation and thermal hydrogenation and evaluated the effects of the machined surfaces (M), nanotubular surfaces (Nano), and hydrogenated nanotubes (H-Nano) on osteogenesis and osseointegration in vitro and in vivo. After incubation of mouse bone marrow mesenchymal stem cells on the samples, we observed improved cell adhesion, alkaline phosphatase activity, osteogenesis-related gene expression, and extracellular matrix mineralization in the H-Nano group compared to the other groups. Subsequent in vivo studies indicated that H-Nano implants promoted rapid new bone regeneration and osseointegration at 4 weeks, which may be attributed to the active osteoblasts adhering to the nanotubular/superhydrophilic surfaces. Additionally, the Nano group displayed enhanced osteogenesis in vitro and in vivo at later stages, especially at 8 weeks. Therefore, we report that hydrogenated superhydrophilic nanotubes can significantly accelerate osteogenesis and osseointegration at an early stage, revealing the considerable potential of this implant modification for clinical applications.

## 1. Introduction

Titanium-based implants have been widely used in oral implantology and craniofacial reconstructions. However, unsatisfactory osseointegration after implantation has become a major issue due to the biological inertness of titanium [1]. Extensive investigation revealed that surface modification of titanium implants may induce rapid and reliable osseointegration [2,3]. Xu et al. [4] used electrochemical etching and anodic oxidation to prepare surfaces with ordered microholes (20 µm in diameter) and titanium dioxide nanotubes (TNTs, 70 nm in diameter), and the bio-inspired micro/nanotextured surface improved osseointegration. In another study, bioinspired polydopamine (PDA)/graphene oxide (GO)/ type I collagen (Col I) (PGC) nanofilms were coated on the Ti-based implant [5]. In vitro evaluations proved that the protein adsorption, biocompatibility, and osteogenic induction ability of PGC nanofilms were significantly enhanced. Furthermore, Wei et al. [6] modified TNTs with bone morphogenetic protein 2/macrophage-derived exosomes. They demonstrated that the functional TNTs remarkably improved the expression of early osteogenic differentiation markers. Among these biomimetic modifications, titanium surfaces with nanotopography and hydrophilic properties are considered to be a simple and promising tool for enhancing osseointegration [7,8,9].

Studies on TNTs revealed their excellent biocompatibility, antibacterial properties, and ability to load and deliver drugs [10,11,12]. Lv et al. [13] reported that TNTs significantly induce the osteogenic differentiation of human adipose-derived stem cells via an epigenetic mechanism. In another study, bone marrow stromal cells cocultured with umbilical vein endothelial cells showed significantly improved vascularization and osteogenic differentiation after incubation with 100 nm diameter TNTs, as compared to pure titanium and 30 nm diameter TNTs [14]. In addition, Yu et al. [15] suggested that TNTs with small diameters exhibit better cell attachment and proliferation, whereas TNTs with large diameters are conducive to osteogenic capability and oxidation resistance.

Although TNTs can effectively promote the osteogenic activity of bone-forming cells or preosteoblasts, it remains unclear whether these inferences are consistent under in vivo conditions. Baker et al. [16] proposed that TNTs can stimulate bone marrow cell differentiation in vitro and promote bone regeneration and bonding strength in rodents. However, Sanchez et al. [17] observed no significant differences between the use of TNTs and polished titanium after 30 days of healing in rodents. Although it was confirmed that TNTs with large diameters are beneficial to cell differentiation, a recent study revealed that the use of 15 nm diameter TNTs had positive effects at the early stage of osseointegration [18]. Thus, additional studies should be conducted to investigate the consistency of the effects of TNTs on bone formation both in vitro and in vivo.

Through superhydrophilic modification, commercially available titanium implants with superhydrophilic surfaces can be used to accelerate osseointegration. Furthermore, the hydrophilicity of titanium implants has attracted increasing attention for shortening osseointegration phages [19,20] and improving in vivo outcomes of systemic diseases affecting bone health [21,22]. Notably, the osteogenic responses to hydrophilic surfaces reportedly improved, as evidenced by the upregulated expression of differentiation markers, earlier deposits of calcified matrix, and increased mineralized bone formation [23,24,25]. A previous study obtained superhydrophilic implant surface by atmospheric-pressure plasma treatment, and confirmed that the superhydrophilic implant can induce high hard-tissue differentiation in vivo [26]. In addition to promoting bone formation, superhydrophilic surfaces can inhibit bacterial adhesion and viability [27].

However, most superhydrophilic surfaces require special storage conditions [28], induction of other elements [29], or ultraviolet light irritation [30]. Henningsen et al. [31] applied UV irradiation or nonthermal plasma (NTP) treatment on titanium to obtain hydrophilic surfaces. They demonstrated that murine osteoblasts on hydrophilic surfaces displayed better cell attachment and growth. However, optimal functionalization of UV irradiation or NTP treatment does not seem feasible under clinical conditions. More importantly, the sustainability of superhydrophilic surfaces is unsatisfactory. A recent experimental study reported that a hydrophilic surface modified via plasma treatment enhanced the differentiation of stem cells and osteoblast-like cells, whereas aging surfaces reduced the efficacy of plasma on cellular responses after 7 days [32].

In the present study, electrochemical anodic oxidation and thermal hydrogenation were conducted to prepare nanostructured/superhydrophilic surfaces. The main advantages of the proposed approaches include the possibility of precisely adjusting the parameters of nanotubes, formation of superhydrophilic surfaces with no impurities introduced [33], and high stability of hydrophilicity in a laboratory environment [34], with no need for wet storage. Our previous study preliminarily evaluated the effects of a nanostructured/superhydrophilic surface on the behavior of an osteoblast-like cell line [35]. However, to predict whether the modified surface can actively influence clinical outcomes, comprehensive in vitro experiments and preclinical investigations are required to extensively assess surface characteristics. Given the role of mesenchymal stem cells in tissue repair and osseointegration [36], this study evaluated the effect of the modified materials on osteogenic differentiation of BMSCs. The present study was conducted considering the controversy regarding the osteogenesis of nanotubes in vitro versus in vivo and the lack of preclinical studies on superhydrophilic nanotubes. Following in vitro studies of the modified nanostructured/superhydrophilic samples employing BMSCs, the osteogenesis and osseointegration abilities of the designed implants with different surfaces were analyzed in a rabbit model.

## 2. Materials and Methods

### 2.1. Sample Preparation

Titanium discs (10 × 10 × 0.2 mm) were used for the in vitro study, whereas cylindrical implants (3.5 × 6 mm) with different surfaces were specially designed for the in vivo study. Pure titanium discs and implants (99.99%; Beijing Cuibolin Nonferrous Technology Development Co. Ltd., Beijing, China) were mechanically polished to produce smooth surfaces and used as the control (M group). The samples were disposed of as previously described [33]. Briefly, machined titanium was prepared via anodic oxidation at 50 V in ethylene glycol (0.5%/10% (*w*/*v*) ammonium fluoride/deionized water) for 15 min and then annealed at 500 °C for 2 h in air to obtain nanotubular surface (Nano group) with titanium dioxide nanotubes (TNTs). Subsequently, the experiments were annealed at 500 °C for 4 h (hydrogen, 0.95 × 10^5^ Pa) to generate the hydrogenated TNTs (H-Nano group). A field-emission scanning electron microscope (S-4800 FESEM; Hitachi, Tokyo, Japan) was used to observe the surface morphology.

### 2.2. In Vitro Studies

#### 2.2.1. Cell Adhesion and Proliferation Assays

Mouse bone mesenchymal stem cells (BMSCs, MUBMX-01001; OriCell, Shanghai, China) were cultured in complete medium (BDMF-03011 and MUXMX-05001; OriCell). A cell counting kit-8 assay (CCK-8; Dojindo Laboratories, Kumamoto, Japan) was conducted to evaluate the adhesion and proliferation activities of BMSCs cultured on different samples. For the proliferation assay, BMSCs were seeded onto the samples (2 × 10^4^ cells/well) and cultured for 1, 3, 5, 7, and 10 days. The results were presented as units of optical density (OD) read at 450 nm. For the cell adhesion assay, BMSCs were incubated on the M, Nano, and H-Nano discs in 24-well plates (1 × 10^5^ cells/well). After culturing for 1, 2, and 4 h, cell adhesion was measured using a CCK-8 assay.

#### 2.2.2. Cell Morphology

Phalloidin was used to evaluate the cytoskeleton and cell morphology. BMSCs were seeded onto the different samples (1 × 10^4^ cells/well) for 24 h. After fixing with 4% paraformaldehyde for 15 min and permeabilizing with Triton X-100, the samples were labelled with Alexa Fluor™ 555 Phalloidin (#8953; Cell Signaling Technology, Danvers, MA, USA) for 1 h in the dark. After rinsing with PBS, the cell nuclei were stained with DAPI (ZLI-9557; ZSGB-BIO, Beijing, China) for 5 min. All samples were observed using a fluorescence microscope (BX51; Olympus, Tokyo, Japan).

#### 2.2.3. Alkaline Phosphatase (ALP) Activity Assay

The BMSCs were seeded onto the samples at a density of 2 × 10^4^ cells/well. After culturing for 3, 5, and 7 days in osteogenic medium (MUXMX-90021; OriCell), the cells were lysed using cell lysis buffer (P0013J; Beyotime, Shanghai, China). ALP activity was detected using BCA protein and ALP assay kits (Beyotime). The measurements were normalized to the total protein concentration. To analyze the direct osteogenic inducibility of the different surfaces, BMSCs were cultured on the M, Nano, and H-Nano discs in complete medium without osteogenic factors for 3 and 5 days. Subsequently, ALP activity was assessed as previously mentioned.

#### 2.2.4. In Vitro Mineralization

Alizarin red S (ARS) staining was performed to evaluate extracellular matrix (ECM) mineralization. After incubation in osteogenic induction medium for 14 and 21 days, the BMSCs on the M, Nano, and H-Nano surfaces were washed and fixed with 4% paraformaldehyde for 15 min. The samples were then stained with ARS solution (pH 4.2; OriCell). A stereoscopic microscope equipped with a digital camera was used to observe and photograph the samples. To quantify ECM calcium accumulation, stained samples were incubated with 330 μL of cetylpyridinium chloride (CPC, 10% *w*/*v*) for 30 min at room temperature. Subsequently, the absorbance was measured at 562 nm using a spectrophotometer (SpectraMax Paradigm; Molecular Devices, Sunny Vale, CA, USA).

#### 2.2.5. Reverse Transcription Polymerase Chain Reaction (RT-PCR)

The BMSCs were incubated on the samples in six-well plates (1 × 10^5^ cells/well) to reach 70–80% confluence, and the complete medium was replaced with osteogenic medium. After incubation for another 14 days, total RNA was extracted using TRIzol reagent (Invitrogen, Carlsbad, CA, USA) and then reverse-transcribed into cDNA using a reagent kit (TaKaRa, Shiga, Japan), following the manufacturer’s instructions. The expression levels of osteogenesis-related genes, specifically *ALP*, type I collagen (*COL-1*), osteocalcin (*OCN*), bone morphogenetic protein 2 (*BMP-2*), and runt-related transcription factor 2 (*RUNX2*), were determined using real-time RT-PCR (CWBio, Beijing, China). The results were normalized using *GAPDH* expression as an internal control. The primer sequences for the target genes (Shenggong, Shanghai, China) are listed in Table 1.

### 2.3. In Vivo Studies

#### 2.3.1. Implants

**Figure 1:**
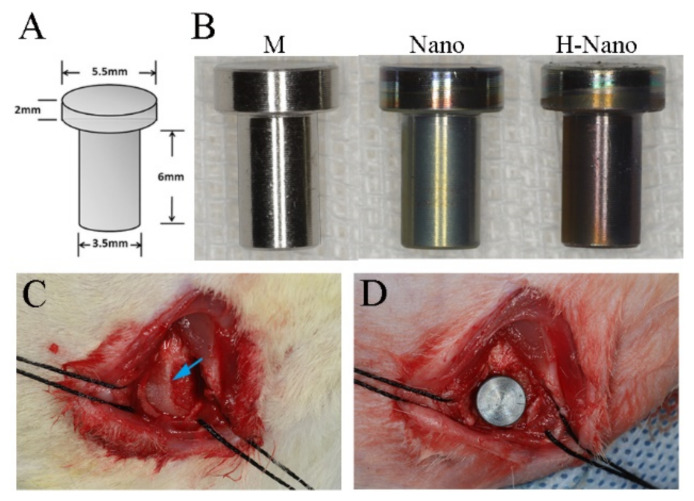
(**A**) The design and specifications of the implant. (**B**) Experimental implants of three groups: machined polished implant (M), anode oxidized implant with nanotubes (Nano), and hydrogenated implant (H-Nano). (**C**) Implantation site. Blue arrow: the distal metaphysis of the femoral condyle. (**D**) Implant placement.

The design and parameters of the implants used in this study are shown in Figure 1A. Pure titanium was processed into implants as required; after mechanical polishing, the M implants were prepared. Subsequently, the M implants were modified as described in Section 2.1. Photographs of the M, Nano, and H-Nano implants are presented in Figure 1B.

#### 2.3.2. Animals

The study design was approved by the Animal Ethical and Welfare Committee of the Beijing Stomatological Hospital, Capital Medical University (Beijing, China). All procedures involving animals followed the guidelines on the care and use of laboratory animals. Eighteen 20 week old adult male New Zealand white rabbits, weighing 2.7–3.2 kg, were used for in vivo experiments (*n* = 6 per group). The animals were acclimatized for 2 weeks before the surgical procedures.

#### 2.3.3. Surgical Procedures

All surgical procedures were performed under general anesthesia using a mixture of xylazine (5 mg/kg) and ketamine (35 mg/kg) via intramuscular administration. The surgical procedures were performed as previously described [37]. In brief, a lateral 1.5 cm femoral condyle incision was made to expose the distal metaphyses (Figure 1C). A bone defect (Φ 3.5 × 6 mm) was created on each lateral femoral condyle using rotary drills under chilled saline. Press-fit placement with an M, Nano, or H-Nano implant based on a computer-generated randomization chart was conducted (Figure 1D). The wound was sutured in two layers. Prophylactic administration of penicillin (four million IU) was performed for 3 days. For double-fluorescence labeling of the bone [38], the rabbits were injected with tetracycline (80 mg/kg; Sigma-Aldrich, St. Louis, MO, USA) at 10 and 11 days before sacrifice, whereas calcein (8 mg/kg; Sigma-Aldrich) was injected at 3 and 4 days before sacrifice. After a healing period of 4 or 8 weeks, the rabbits were sacrificed to harvest the bone blocks containing the implants.

#### 2.3.4. Micro-Computed Tomography (Micro-CT)

Micro-CT (100 kv, 38 μA, SkyScan1276; Bruker, Billerica, MA, USA) was conducted to detect the femoral condyle blocks. For each sample, a threshold value and regions of interest (ROIs) were chosen, and images were reconstructed and analyzed using CT-Analyser (CTAn) software (Bruker). The bone volume/total volume fraction (BV/TV, %), trabecular bone number (Tb *n*, 1/mm), and Tb thickness (Tb Th, mm) were subsequently measured.

#### 2.3.5. Histology

After fixation with 10% formalin for 7 days, the blocks were gradually dehydrated in a series of ethanol solutions (70–100%), embedded in methacrylate-based resin, and longitudinally sectioned, aiming for the center of the titanium implants along their long axes. Each section was ground to approximately 200 μm thickness and polished to a final thickness of approximately 40 μm. Tetracycline and calcein fluorescence were observed using a fluorescence microscope. After light irradiation, tetracycline appeared yellow, whereas calcein appeared green. To evaluate new bone regeneration, the mineral apposition rate (MAR, μm/d) was determined by measuring the distance between two fluorescence markers using ImageJ software (NIH, Bethesda, MD, USA).

Subsequently, all slices were stained with methylene blue/acid fuchsin and observed using light microscopy (BX51; Olympus).

#### 2.3.6. Histometric Analysis

Histomorphometry was performed using ImageJ software (NIH). The percentage of direct contact between the bone and implant surface (BIC%) and the bone fraction (BF%) at a distance of 0.5 mm around the implant were calculated.

### 2.4. Statistical Analysis

Quantitative data were presented as the means ± SD. The data were analyzed via one-way analysis of variance (ANOVA) or Kruskal–Wallis test using SPSS software (version 19; SPSS, Chicago, IL, USA). The results were considered statistically significant at *p* < 0.05.

## 3. Results

### 3.1. In Vitro Studies

#### 3.1.1. Surface Characteristics

**Figure 2:**
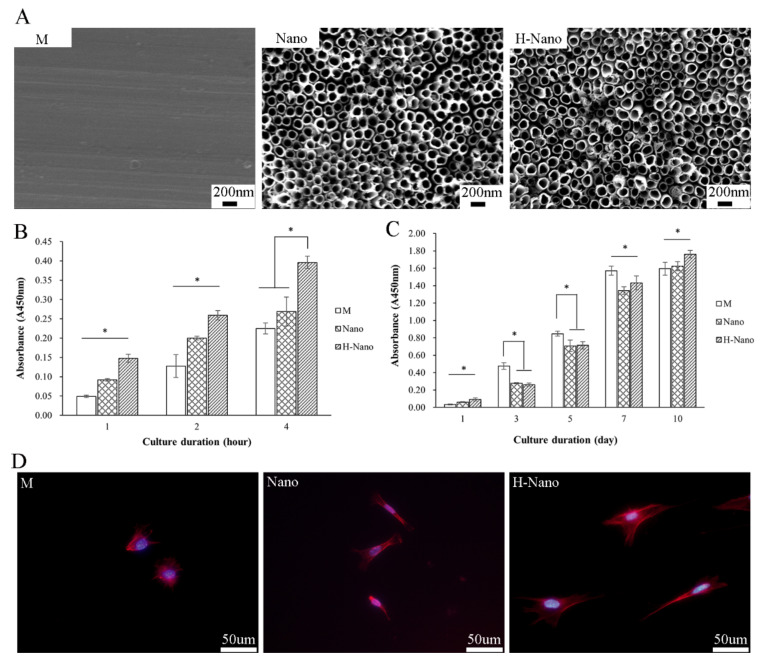
(**A**) SEM images of the M, Nano, and H-Nano surfaces at magnification of ×30,000. (**B**) BMSC adhesion on the surfaces of the M, Nano, and H-Nano detected by cell counting kit-8 assay after culturing for 1, 2, and 4 h. (**C**) Cell proliferation activity on specimens after 1, 3, 5, 7, and 10 days of incubation. (**D**) Immunofluorescent images of BMSCs adhered to the M, Nano, and H-Nano surfaces after culturing for 24 h. Cellular F-actin was labeled by Alexa Fluor™ 555 Phalloidin (red) and nuclei were stained with DAPI (blue). * *p* < 0.05.

The machined titanium exhibited a relatively smooth surface with regular mechanically polished scratches (Figure 2A). In the Nano and H-Nano groups, a uniformly arranged nanotopography with approximately 100 nm diameter nanotubes was observed on the surface.

#### 3.1.2. BMSC Adhesion and Proliferation

The number of adherent cells on the H-Nano surface was significantly higher than that on the other surfaces after 1, 2, and 4 h of incubation (*p* < 0.05; Figure 2B). In addition, cell adhesion on the Nano surface at 1 and 2 h was significantly greater than that on the M surface (*p* < 0.05). These results demonstrate that the nanotubes, especially those with superhydrophilic surfaces, are more conducive to the early adherence of BMSCs.

After 1 day, the cell number was significantly higher in the H-Nano group than in the M and Nano groups (Figure 2C). After 3, 5, and 7 days, cell proliferation in the M group was significantly increased compared to the other groups (*p* < 0.05). However, after 10 days, the BMSCs in the M group barely proliferated, whereas the cells in the Nano and H-Nano groups showed a significantly better proliferation ability than those in the M group (*p* < 0.05). These results indicate that, although the M surface promotes BMSC proliferation at the early stage, the nanotubular and superhydrophilic surfaces improve BMSC proliferation at the later stage.

Further observation of cell morphology on the different surfaces revealed that most BMSCs in the M group presented as spheres with poorly organized cytoskeletons (Figure 2D). By contrast, BMSCs in the Nano group showed greatly spread morphologies with pseudopodia, whereas cells in the H-Nano group displayed widely flattened and spindle-shaped cytoplasm with a large spreading area and prominent cytoskeletons.

#### 3.1.3. Osteogenic Differentiation of BMSCs

**Figure 3:**
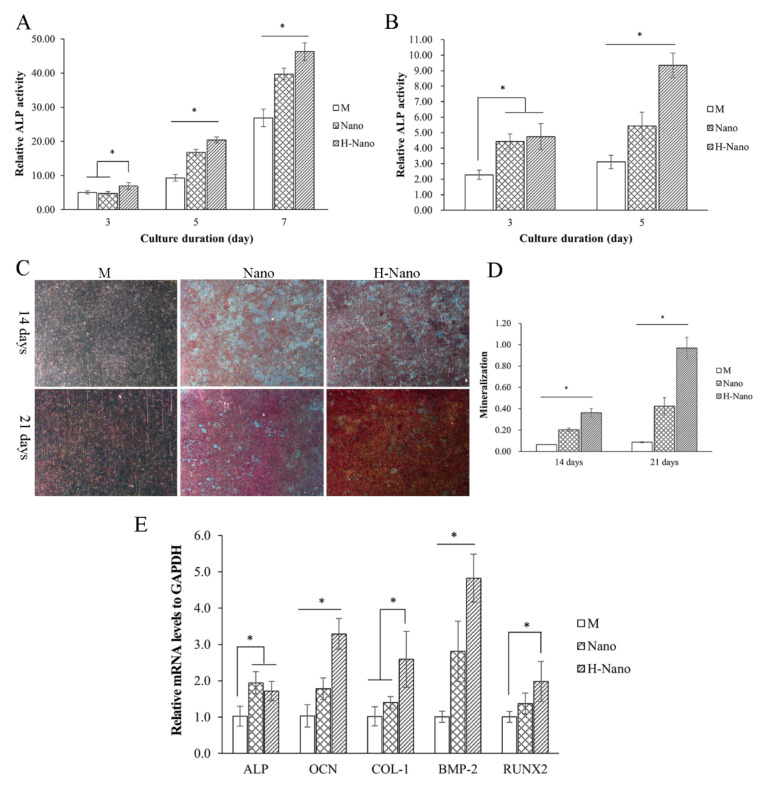
(**A**) Relative alkaline phosphatase (ALP) activity of BMSCs on the M, Nano, and H-Nano surfaces cultured in the osteogenic medium for 3, 5, and 7 days. (**B**) Relative ALP activity of BMSCs on the M, Nano, and H-Nano surfaces without osteogenic factors. (**C**) Alizarin red S staining for calcium nodules after incubation on the specimens for 14 and 21 days. (**D**) Quantitative analysis of extracellular matrix mineralization on the M, Nano, and H-Nano surfaces at 14 and 21 days. (**E**) Relative expression levels of osteogenesis-related genes of BMSCs cultured on specimens for 14 days. Expression of *ALP*, osteocalcin (*OCN*), type I collagen (*COL-1*), bone morphogenetic protein 2 (*BMP-2*), and runt-related transcription factor 2 (*RUNX2*) is presented according to the relative amount of mRNA with the formula 2^(−∆∆Ct)^. * *p* < 0.05.

After culturing in osteogenic medium for 5 and 7 days, BMSCs in the Nano group exhibited higher ALP activity than in the M group (*p* < 0.05; Figure 3A). Compared to other groups, the ALP activity of cells on the H-Nano with a superhydrophilic surface was remarkably improved at 3, 5, and 7 days (*p* < 0.05).

To further evaluate the direct osteogenic inducibility of the different surfaces, BMSCs were incubated on samples without osteogenic factors. After 3 and 5 days, BMSCs in the Nano and H-Nano groups exhibited significantly increased ALP activity compared to the M group (Figure 3B). Furthermore, at 5 days, the ALP activity of the BMSCs in the H-Nano group was higher than that in the Nano group (*p* < 0.05). These results suggest that nanostructures, especially superhydrophilic surfaces, can stimulate the early osteogenic differentiation of BMSCs even in the absence of osteogenic factors.

ARS staining of BMSCs at 14 days revealed the obvious mineralized calcium deposits in the Nano and H-Nano groups (Figure 3C). At 21 days, the calcium nodules were more distinctly observed in the H-nano group than in the M and Nano groups. In addition, the quantitative mineralized calcium deposition was significantly increased in the Nano and H-Nano groups compared to the M control (Figure 3D). Notably, BMSCs in the H-Nano group showed the highest degree of ECM mineralization among the experimental groups, suggesting that the use of an H-Nano surface is the most effective in promoting osteogenic differentiation.

#### 3.1.4. Osteogenic-Related Gene Expression

After 14 days, the H-Nano group exhibited an upregulation of all target genes compared to the M group (*p* < 0.05; Figure 3E). In addition, the expression of *ALP*, *OCN*, and *BMP-2* was significantly different between the Nano and M groups. Furthermore, significant differences in *COL-1*, *OCN*, and *BMP-2* expression levels were observed between the H-Nano and Nano groups (*p* < 0.05). Notably, *ALP* expression was slightly lower in the H-Nano group than in the Nano group.

### 3.2. In Vivo Studies

#### 3.2.1. Micro-CT Analysis

**Figure 4:**
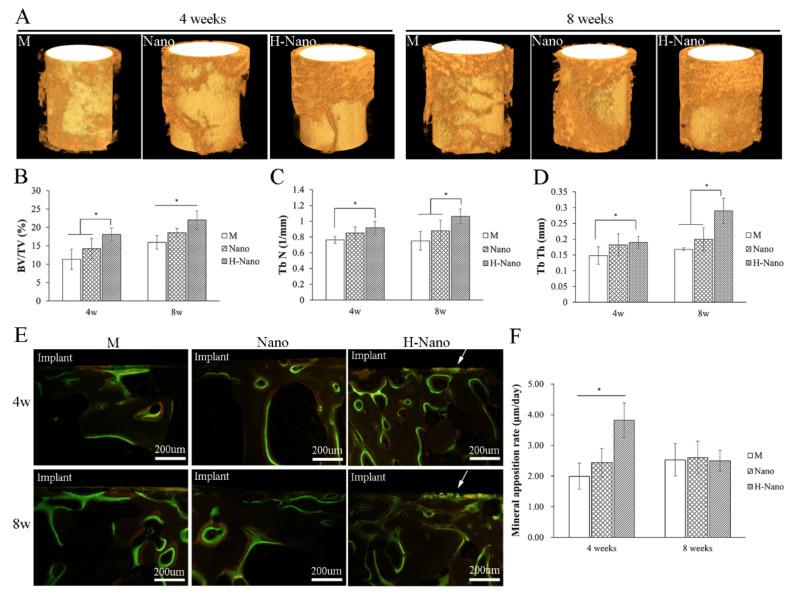
(**A**) Rendered 3D microcomputed tomography images of bone tissue around the M, Nano, and H-Nano implants after 4 and 8 weeks of healing. (**B**) Bone volume/total volume fraction (BV/TV, %), (**C**) trabecular bone number (Tb N), and (**D**) trabecular bone thickness (Tb Th) of three groups obtained from quantitative analysis of the Micro-CT data. (**E**) Double-labeled fluorescent images of the M, Nano, and H-Nano group at 4 and 8 weeks. The fluorescent yellow and green strips represent new bone apposition areas labeled by tetracycline and calcein, respectively. White arrows: contact osteogenesis occurred on the implant surface. (**F**) Quantification of the bone mineral apposition rate in the fluorescence labeled samples. * *p* < 0.05.

Representative reconstructed 3D images of unified regions of interest around the implants based on micro-CT data were generated (Figure 4A). The BV/TV, Tb *n*, and Tb Th values of the peri-implant bone are presented in Figure 4B–D. All parameters obtained for the H-Nano group were significantly higher than those for the M group (*p* < 0.05) at both timepoints, whereas there was no significant difference between the M and Nano groups except for the BV/TV after 8 weeks of healing. Furthermore, the BV/TV of the H-Nano group was significantly higher than that of the Nano group from 4 to 8 weeks (*p* < 0.05), whereas the Tb *n* and Tb Th showed significant differences after 8 weeks (*p* < 0.05). This implies that the maturation of the Tb may have occurred at the late stage of bone regeneration.

#### 3.2.2. MAR Measurement

Fluorescent yellow (labeled by tetracycline) and green (labeled by calcein) strips were considered as mineral apposition zones (Figure 4E). Green fluorescence near the implant surface indicated that contact osteogenesis occurred in the H-Nano group. Significant differences were observed among all groups at 4 weeks, in which the H-Nano group exhibited the highest MAR value (*p* < 0.05; Figure 4F). Although the MAR of the H-Nano group decreased at 8 weeks, it was comparable to that of the other groups. Our results demonstrate that the H-Nano group had the fastest bone regeneration.

#### 3.2.3. Histological Examination

**Figure 5:**
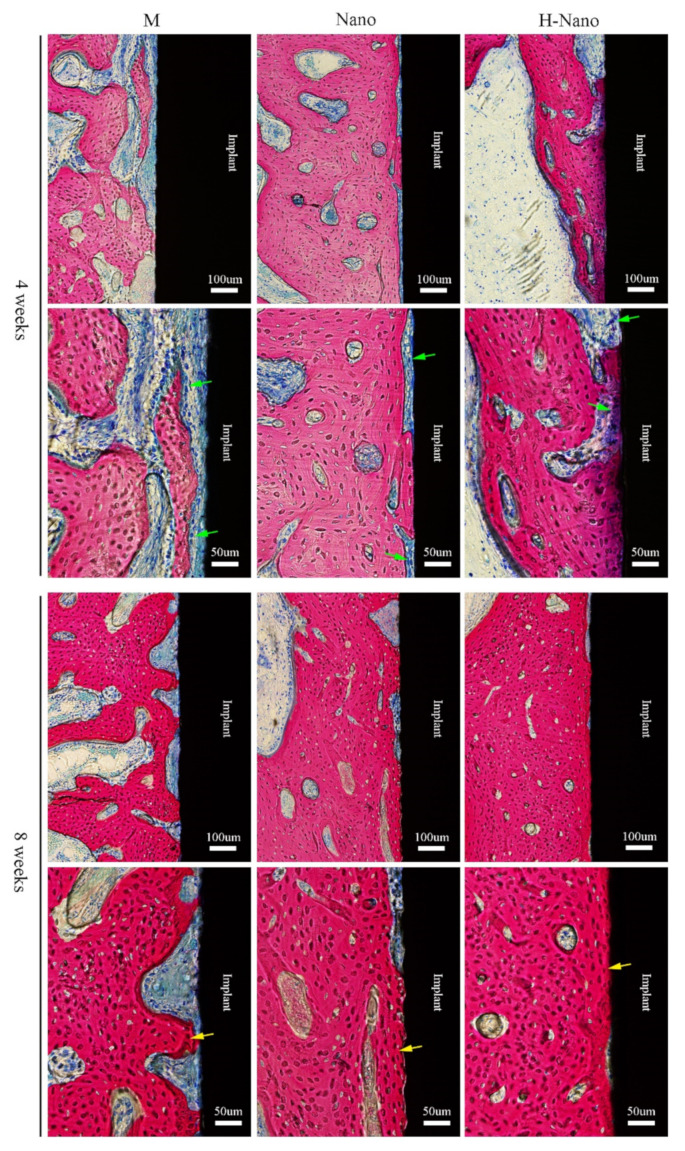
Histological observation of the M, Nano, and H-Nano group after 4 and 8 weeks. Each set of images for all groups were illustrated from three different magnifications of the same region. Methylene blue and acid fuchsin staining. Green arrows: osteoblasts (dark blue). Yellow arrows: new bone formation (dark red).

The implants, bone matrix, new bone, osteoid, and osteoblasts were stained black, red, dark red, blue-gray, and dark blue, respectively (Figure 5). Although all groups exhibited new bone regeneration at 4 and 8 weeks, the H-Nano implants displayed better and faster peri-implant osseointegration than the other groups.

At 4 weeks, obvious gaps were observed at the bone–implant interface in the M group, with many osteoclasts around the original bone than the implant, suggesting that distant osteogenesis was predominant. In the Nano and H-Nano groups, bone–implant contact was more obvious than that in the M group. In addition to osteoblasts on the initial bone side, many osteoblasts were observed near the Nano and H-Nano surfaces, indicating that both contact and distant osteogenesis occurred.

After 8 weeks of healing, a newly formed bone matrix (dark red) achieved greater osseointegration around the M implant, and an osteoid was observed between the implant and bone. In the H-Nano group, the bone closely contacted with the implants with no obvious gaps and osteoblasts, demonstrating that peri-implant osseointegration had matured. Notably, the osteogenic efficiency and osseointegration of the H-Nano group were the highest among all groups.

#### 3.2.4. Histometric Assessment

**Figure 6:**
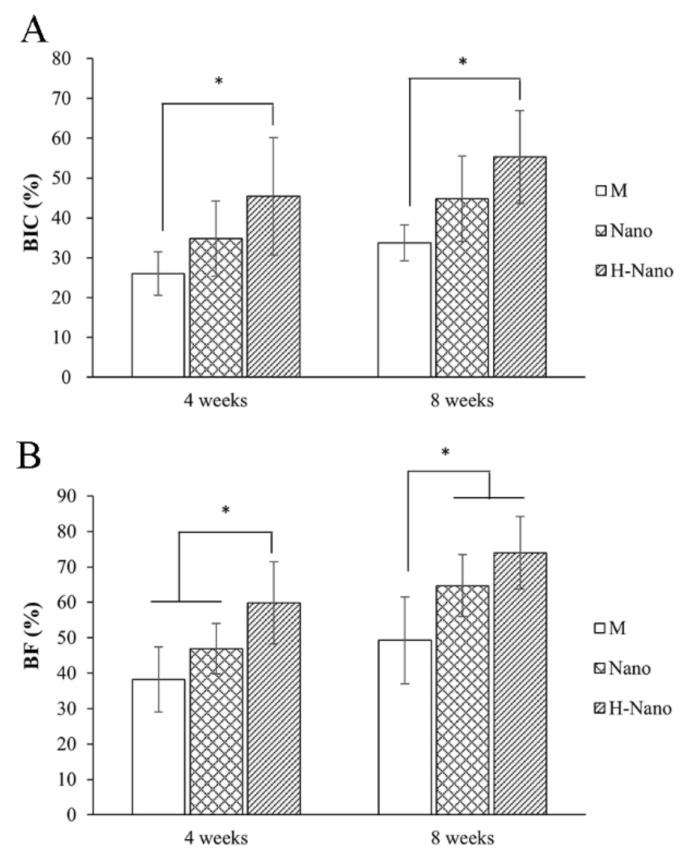
Quantification of (**A**) the bone–implant contact (BIC%) and (**B**) bone fraction (BF%) in the histological samples. * *p* < 0.05.

After 4 and 8 weeks of healing, the BIC% and BF% values in the H-Nano group were significantly higher than those in the M group (*p* < 0.05; Figure 6A,B). In addition, significant differences in BF% values were observed between the M and Nano groups at 8 weeks (*p* < 0.05).

## 4. Discussion

This study was designed to comprehensively assess the positive effects of a nanotubular/superhydrophilic surface during the early stage of osseointegration using in vitro and in vivo experiments. Cell adhesion, proliferation, ALP activity, ECM mineralization, and gene expression assays were performed in vitro to evaluate the osteogenic differentiation of BMSCs. Moreover, in vivo early bone regeneration and osseointegration were investigated in a rabbit femur implant model using micro-CT, new bone deposition velocity, and histological and histometric analyses. Our findings confirmed that hydrogenated TNTs with superhydrophilic surfaces can promote early osteogenic differentiation in vitro and accelerate osteogenesis and osseointegration in vivo, whereas the nanotubes mainly influenced new bone formation at the late stage of healing.

New bone formation proceeds in a structural osteoclast resorption pit preconditioned by osteoclast-mediated bone resorption, leading to features in micro-, meso-, and nanoscale dimensions [39]. Therefore, biomimetic topography has been investigated to improve osseointegration [2]. Among these, nanotopography is a critical factor in surface modification. In this study, TNTs fabricated via electrochemical anodic oxidation were used as nanostructures to mimic the osteogenic environment. TNTs formed in ethylene glycol (a viscous organic electrolyte) were homogeneous due to the relatively low rate of chemical dissolution and fluoride ion transmission [40]. Our results revealed that TNTs promoted BMSC adhesion with spread morphologies, and many pseudopodia (Figure 2D). Subsequently, the ALP activity and EMC mineralization of BMSCs in the Nano group increased compared to that in the control. Previous studies reported that TNTs could directly regulate stem cells or osteoblasts through extracellular mechanical signals, followed by transmission into intracellular signaling pathways [3,41,42] or epigenetic regulation [13,43], which eventually leads to superior osteogenic differentiation [44].

Additionally, as we previously reported in more detail [33], the H-Nano group presented a superhydrophilic surface with a water contact angle of 3.5°. In the meantime, our previous study confirmed that the superhydrophilicity of hydrogenated TNTs was relatively stable (angle variation of 5° to 8° within 4 weeks) [34]. In another study, stable hydrophilicity was achieved by reactive plasma modification and wet storage, which led to improved cell adhesion, viability, and ALP activity [45]. Tang et al. [46] used UV irradiation to greatly improve the hydrophilicity of the TiO_2_ coating on zirconia implant, and in vitro assessments displayed the enhanced ALP activity, mineralization, and osteogenesis-related genes expression. In specific, the H-Nano group with superhydrophilic surfaces in this study displayed optimal cell attachment and cell functions, including ALP synthesis, ECM mineralization, and osteogenesis-related gene expression, compared to the M and Nano groups. Additionally, cell migration and aggregation on the titanium implant with a superhydrophilic surface were distinctly observed in vivo (Figure 5). These results suggest that surface wettability is strongly associated with cell adhesion, differentiation, and bone regeneration and may be an even more important factor than surface topography, which is consistent with another study [47]. Interestingly, the underlying mechanisms may be related to protein adsorption. During osseointegration, the superhydrophilic surface promotes the preferential adsorption of adhesion-related proteins (e.g., fibronectin and vitronectin) [48,49], which consequently leads to early cell adhesion, differentiation, and osseointegration.

Notably, the number of proliferated BMSCs on the Nano and H-Nano surfaces was lower than that on the M surface at 3, 5, and 7 days. We speculated that the BMSCs on the Nano and H-Nano surfaces underwent mild differentiation during proliferation, resulting in a low cell count at the early stage. This was confirmed by the ALP activity assay results using a medium without osteogenic factors (Figure 3B), indicating the direct osteogenic induction potential of nanotopography and wettability. Similarly, Gu et al. [24] reported a decreased proliferation of MC3T3-E1 cells in sand-blasted, large-grit, acid-etched (SLA) surface, and modified SLA (modSLA) surface compared to polished titanium, which coincided with the increased expression levels of differentiation markers. In addition, the H-Nano group had the highest cell numbers after 1 day of incubation, which may represent the high adhesive cell number in the initial time. Furthermore, BMSC proliferation on the Nano and H-Nano surfaces increased after 10 days. Likewise, Klein et al. [50] observed further proliferation of human osteogenic cells on hydrophilic surfaces during the late stage of incubation. Overall, there may be a balance between cell proliferation and differentiation [4] on the different TNT surfaces. Specifically, the general effects of TNTs, especially those with superhydrophilic surfaces, is to promote BMSC differentiation.

ALP is a known marker at the early stage of osteogenic differentiation; its expression and activity are increased during the maturation of the osteogenic lineage. In this study, BMSCs incubated on the H-Nano surfaces exhibited increased ALP activity compared to those on the M and Nano surfaces after 3, 5, and 7 days, whereas *ALP* expression decreased in the H-Nano group after 14 days. These results revealed the early osteogenic differentiation of BMSCs on superhydrophilic surfaces. Importantly, we observed the critical increase of *BMP-2* and *RUNX2* expression in the H-Nano group, indicating active osteogenic transcription and differentiation [51,52]. In addition, upregulated *COL-1* and *OCN* expression in the H-Nano group promotes the initial formation of the bone matrix and regulation of the hard tissue mineralization [53]. As expected, ECM mineralization in the H-Nano group displayed the highest measurements in the late phase of BMSC osteogenic differentiation (Figure 3D). These in vitro experiments validate that the superhydrophilic surfaces accelerate osteogenic differentiation and improve the bone formation potential of BMSCs.

The In vivo investigation using a rabbit model further revealed the enhanced early osseointegration capability of superhydrophilic surfaces. Our MAR analysis indicated significant differences among all groups (H-Nano > Nano > M) at the early stage of healing (Figure 4F). Fluorescent images showed active osteogenesis, especially contact osteogenesis on the superhydrophilic surfaces, whereas contact osteogenesis was barely observed in the control with predominant distant osteogenesis (Figure 4E). Furthermore, histometric results showed that the BIC% value was markedly increased in the H-Nano group compared to the M group (Figure 6A). These results strongly suggest that rapid new bone deposition and early osseointegration activity occurred in superhydrophilic implants.

The osteogenic activity of osteoblasts in vivo may regulate bone metabolism and osseointegration [54,55]. Importantly, our histological analysis showed that more osteoblasts were observed adjacent to the superhydrophilic group than in the control group (Figure 5). Thus, we hypothesized that the active osteoblasts adherent to the H-Nano implant accelerated the MAR. Furthermore, it is notable that the adhesion and differentiation of osteoblasts on modified implant surfaces with osteoconductive characteristics may induce contact osteogenesis [38,56,57]. Khosravi et al. [58] investigated the earliest phases of peri-implant bone healing using intravital optical microscopy and found that nanostructured implant surfaces profoundly influenced the recruitment of endothelial and osteoprogenitor cells, which is beneficial for contact osteogenesis. In addition, hydrophilic surfaces are regarded as conducive for bone formation through the rapid spread of serum to obtain a uniform coating of bioactive molecules, subsequently promoting early cell adhesion, proliferation, and differentiation [48,59,60]. In our study, the superhydrophilic surface may have induced the initial recruitment, attachment, and osteogenic differentiation of osteoblasts in vivo and eventually improved contact osteogenesis and early osteointegration, corroborating the results of in vitro studies.

In addition, we used micro-CT to assess new BV and Tb density around the implants. The BV/TV, Tb Th, and Tb *n* values were higher in the H-Nano group than those in the M and Nano groups, especially at 8 weeks (Figure 4B–D). Subsequent histometric analysis also demonstrated a higher BF% value in the H-Nano group (Figure 6B), suggesting that new bone regeneration surrounding the H-Nano implants was greatly enhanced.

In the meantime, our in vivo results exhibited consistency with the in vitro studies for nanotopography. The Nano group showed higher MAR value at 4 weeks compared with the M group (*p* < 0.05). Additionally, the BV/TV and BF% of Nano implants were significantly different with those of M controls after 8 weeks of healing (Nano > M). The assessments for trabecular bone and BIC were greater in Nano group than those in M group, although the differences failed to reach statistical significance. Our results suggested that the nanotubular morphology could promote osteogenesis in vitro and in vivo, particularly in new bone formation at late phage of healing. In addition, a previous study demonstrated that TiO_2_ nanotubes can greatly accelerate the rate of mineralization and eventually improve bone bonding by up to ninefold [11].

Given that osteointegration is an extremely complicated process caused by a series of biological events, the effects of the nanotubular/superhydrophilic surface on different stages, such as initial protein adoption in the serum, blood coagulation, osteoimmunomodulation, and bone remodeling, should be thoroughly investigated in the future. Furthermore, the specific mechanisms for the enhanced osteogenesis and osteointegration of the modified surface remain unclear. Thus, further studies on elucidating the osteointegration process and underlying molecular mechanisms are required for the potential clinical applications of the nanotubular/superhydrophilic surfaces.

## 5. Conclusions

The in vitro study revealed that the use of titanium implants with nanotubular/superhydrophilic surfaces can considerably improve the cell adhesion and osteogenic differentiation of BMSCs. Moreover, the H-Nano implants can accelerate new bone regeneration and induce excellent osseointegration in vivo at the early stage. Additionally, Nano morphology can enhance osteogenesis both in vitro and in vivo, specifically at the late phase of healing. Our study demonstrated that creating nanotubular/superhydrophilic surfaces in titanium implants is a promising modification method with potential clinical applications.

## Figures and Tables

**Table 1:** Primer pairs used for gene expression analysis.

Gene	Forward Primer (5′ to 3′)	Reverse Primer (5′ to 3′)
*ALP*	GAACAGAACTGATGTGGAATACGAA	CAGTGCGGTTCCAGACATAGTG
*COL-1*	GATGTTGAACTTGTTGTTGCTGAGGG	GGCAGGCGAGATGGCTTATT
*OCN*	TTCTGCTCACTCTGCTGACC	ACCACTCCAGCACAACTCCT
*BMP-2*	TCCCCAGTGACGAGTTTCTC	GTCGAAGCTCTCCCACTGAC
*RUNX2*	GCCGGGAATGATGAGAACTA	GGACCGTCCACTGTCACTTT
*GAPDH*	TGTGTCCGTCGTGGATCTGA	TTGCTGTTGAAGTCGCAGGAG

## Data Availability

The data presented in this study are available in the article.

## References

[1] Souza J.C.M., Sordi M.B., Kanazawa M., Ravindran S., Henriques B., Silva F.S., Aparicio C., Cooper L.F. (2019). Nano-scale modification of titanium implant surfaces to enhance osseointegration. Acta Biomater..

[2] Berger M.B., Slosar P., Schwartz Z., Cohen D.J., Goodman S.B., Anderson P.A., Boyan B.D. (2022). A review of biomimetic topographies and their role in promoting bone formation and osseointegration: Implications for clinical use. Biomimetics.

[3] Chen S., Guo Y., Liu R., Wu S., Fang J., Huang B., Li Z., Chen Z., Chen Z. (2018). Tuning surface properties of bone biomaterials to manipulate osteoblastic cell adhesion and the signaling pathways for the enhancement of early osseointegration. Colloids Surf. B Biointerfaces.

[4] Xu Z., Huang J., He Y., Su J., Xu L., Zeng X. (2022). Fabrication of an ordered micro-/nanotextured titanium surface to improve osseointegration. Colloids Surf B Biointerfaces.

[5] Xu K., Zhou M., Chen W., Zhu Y., Wang X., Zhang Y., Zhang Q. (2021). Bioinspired polydopamine/graphene oxide/collagen nanofilms as a controlled release carrier of bioactive substances. Chem. Eng. J..

[6] Wei F., Li M., Crawford R., Zhou Y., Xiao Y. (2019). Exosome-integrated titanium oxide nanotubes for targeted bone regeneration. Acta Biomater..

[7] Hou C., An J., Zhao D., Ma X., Zhang W., Zhao W., Wu M., Zhang Z., Yuan F. (2022). Surface modification techniques to produce micro/nano-scale topographies on Ti-based implant surfaces for improved osseointegration. Front. Bioeng. Biotechnol..

[8] Vordemvenne T., Wahnert D., Koettnitz J., Merten M., Fokin N., Becker A., Büker B., Vogel A., Kronenberg D., Stange R. (2020). Bone regeneration: A novel osteoinductive function of spongostan by the interplay between its nano- and microtopography. Cells.

[9] Higuchi J., Klimek K., Wojnarowicz J., Opalinska A., Chodara A., Szalaj U., Dąbrowska S., Fudala D., Ginalska G. (2022). Electrospun membrane surface modification by sonocoating with HA and ZnO:Ag nanoparticles-Characterization and evaluation of osteoblasts and bacterial cell behavior in vitro. Cells.

[10] Hosseinpour S., Nanda A., Walsh L.J., Xu C. (2021). Microbial decontamination and antibacterial activity of nanostructured titanium dental implants: A narrative review. Nanomaterials.

[11] Su E.P., Justin D.F., Pratt C.R., Sarin V.K., Nguyen V.S., Oh S., Jin S. (2018). Effects of titanium nanotubes on the osseointegration, cell differentiation, mineralisation and antibacterial properties of orthopaedic implant surfaces. Bone Jt. J..

[12] Jayasree A., Ivanovski S., Gulati K. (2021). ON or OFF: Triggered therapies from anodized nano-engineered titanium implants. J. Control. Release.

[13] Lv L., Liu Y., Zhang P., Zhang X., Liu J., Chen T., Su P., Li H., Zhou Y. (2015). The nanoscale geometry of TiO_2_ nanotubes influences the osteogenic differentiation of human adipose-derived stem cells by modulating H3K4 trimethylation. Biomaterials.

[14] Wu Z., Wang S., Chang J., Huan Z., Li H. (2018). TiO_2_ nanotubes enhance vascularization and osteogenic differentiation through stimulating interactions between bone marrow stromal cells and endothelial cells. J. Biomed. Nanotechnol..

[15] Yu Y., Shen X., Luo Z., Hu Y., Li M., Ma P., Ran Q., Dai L., He Y., Cai K. (2018). Osteogenesis potential of different titania nanotubes in oxidative stress microenvironment. Biomaterials.

[16] Baker E.A., Vara A.D., Salisbury M.R., Fleischer M.M., Baker K.C., Fortin P.T., Roberts R.V., Friedrich C.R. (2020). Titania nanotube morphologies for osseointegration via models of in vitro osseointegrative potential and in vivo intramedullary fixation. J. Biomed. Mater. Res. Part B Appl. Biomater..

[17] Gomez Sanchez A., Katunar M.R., Pastore J.I., Tano de la Hoz M.F., Cere S. (2021). Evaluation of annealed titanium oxide nanotubes on titanium: From surface characterization to in vivo assays. J. Biomed. Mater. Res. A.

[18] Bai L., Zhao Y., Chen P., Zhang X., Huang X., Du Z., Crawford R., Yao X., Tang B., Hang R. (2021). Targeting early healing phase with titania nanotube arrays on tunable diameters to accelerate bone regeneration and osseointegration. Small.

[19] Lang N.P., Salvi G.E., Huynh-Ba G., Ivanovski S., Donos N., Bosshardt D.D. (2011). Early osseointegration to hydrophilic and hydrophobic implant surfaces in humans. Clin. Oral Implant. Res..

[20] Sartoretto S.C., Alves A., Zarranz L., Jorge M.Z., Granjeiro J., Calasans-Maia M. (2017). Hydrophilic surface of Ti6Al4V-ELI alloy improves the early bone apposition of sheep tibia. Clin. Oral Implant. Res..

[21] Cabrera-Domínguez J.J., Castellanos-Cosano L., Torres-Lagares D., Pérez-Fierro M., Machuca-Portillo G. (2019). Clinical performance of titanium-zirconium implants with a hydrophilic surface in patients with controlled type 2 diabetes mellitus: 2-year results from a prospective case-control clinical study. Clin. Oral Investig..

[22] Siqueira R., Ferreira J.A., Rizzante F.A.P., Moura G.F., Mendonça D.B.S., de Magalhães D., Cimões R., Mendonça G. (2021). Hydrophilic titanium surface modulates early stages of osseointegration in osteoporosis. J. Periodontal Res..

[23] Wall I., Donos N., Carlqvist K., Jones F., Brett P. (2009). Modified titanium surfaces promote accelerated osteogenic differentiation of mesenchymal stromal cells in vitro. Bone.

[24] Gu Y.-X., Du J., Si M.-S., Mo J.-J., Qiao S.-C., Lai H.-C. (2013). The roles of PI3K/Akt signaling pathway in regulating MC3T3-E1 preosteoblast proliferation and differentiation on SLA and SLActive titanium surfaces. J. Biomed. Mater. Res. A.

[25] Bandyopadhyay A., Shivaram A., Mitra I., Bose S. (2019). Electrically polarized TiO_2_ nanotubes on Ti implants to enhance early-stage osseointegration. Acta Biomater..

[26] Tsujita H., Nishizaki H., Miyake A., Takao S., Komasa S. (2021). Effect of plasma treatment on titanium surface on the tissue surrounding implant material. Int. J. Mol. Sci..

[27] Shao H., Ma M., Wang Q., Yan T., Zhao B., Guo S., Tong S. (2022). Advances in the superhydrophilicity-modified titanium surfaces with antibacterial and pro-osteogenesis properties: A review. Front. Bioeng. Biotechnol..

[28] Hyzy S.L., Olivares-Navarrete R., Ortman S., Boyan B.D., Schwartz Z. (2017). Bone morphogenetic protein 2 alters osteogenesis and anti-inflammatory profiles of mesenchymal stem cells induced by microtextured titanium in vitro. Tissue Eng. Part A.

[29] Han Y., Zhou J., Zhang L., Xu K. (2011). A multi-scaled hybrid orthopedic implant: Bone ECM-shaped Sr-HA nanofibers on the microporous walls of a macroporous titanium scaffold. Nanotechnology.

[30] Leon-Ramos J.R., Diosdado-Cano J.M., Lopez-Santos C., Barranco A., Torres-Lagares D., Serrera-Figallo M. (2019). Influence of titanium oxide pillar array nanometric structures and ultraviolet irradiation on the properties of the surface of dental implants—A pilot study. Nanomaterials.

[31] Henningsen A., Smeets R., Hartjen P., Heinrich O., Heuberger R., Heiland M., Precht C., Cacaci C. (2018). Photofunctionalization and non-thermal plasma activation of titanium surfaces. Clin. Oral Investig..

[32] Berger M.B., Bosh K.B., Cohen D.J., Boyan B.D., Schwartz Z. (2021). Benchtop plasma treatment of titanium surfaces enhances cell response. Dent. Mater..

[33] Wang C., Wang X., Lu R., Gao S., Ling Y., Chen S. (2021). Responses of human gingival fibroblasts to superhydrophilic hydrogenated titanium dioxide nanotubes. Colloids Surf B Biointerfaces.

[34] Gao S., Lu R., Wang X., Chou J., Wang N., Huai X., Wang C., Zhao Y., Chen S. (2020). Immune response of macrophages on super-hydrophilic TiO_2_ nanotube arrays. J. Biomater. Appl..

[35] Lu R., Wang C., Wang X., Wang Y., Wang N., Chou J., Li T., Zhang Z., Ling Y., Chen S. (2018). Effects of hydrogenated TiO_2_ nanotube arrays on protein adsorption and compatibility with osteoblast-like cells. Int. J. Nanomed..

[36] Soudi A., Yazdanian M., Ranjbar R., Tebyanian H., Yazdanian A., Tahmasebi E., Keshvad A., Seifalian A. (2021). Role and application of stem cells in dental regeneration: A comprehensive overview. EXCLI J..

[37] Salou L., Hoornaert A., Louarn G., Layrolle P. (2015). Enhanced osseointegration of titanium implants with nanostructured surfaces: An experimental study in rabbits. Acta Biomater..

[38] Zhao B., Li X., Xu H., Jiang Y., Wang D., Liu R. (2020). Influence of simvastatin-strontium-hydroxyapatite coated implant formed by micro-arc oxidation and immersion method on osteointegration in osteoporotic rabbits. Int. J. Nanomed..

[39] Boyan B.D., Lotz E.M., Schwartz Z. (2017). Roughness and hydrophilicity as osteogenic biomimetic surface properties. Tissue Eng. Part A.

[40] Khudhair D., Bhatti A., Li Y., Hamedani H.A., Garmestani H., Hodgson P., Nahavandi S. (2016). Anodization parameters influencing the morphology and electrical properties of TiO_2_ nanotubes for living cell interfacing and investigations. Mater. Sci. Eng. C Mater. Biol. Appl..

[41] Oh S., Brammer K.S., Li Y.S.J., Teng D., Engler A.J., Chien S., Jin S. (2009). Stem cell fate dictated solely by altered nanotube dimension. Proc. Natl. Acad. Sci. USA.

[42] Tong Z., Liu Y., Xia R., Chang Y., Hu Y., Liu P., Zhai Z., Zhang J., Li H. (2020). F-actin regulates osteoblastic differentiation of mesenchymal stem cells on TiO_2_ nanotubes through MKL1 and YAP/TAZ. Nanoscale Res. Lett..

[43] Liu Y., Tong Z., Wang C., Xia R., Li H., Yu H., Jing J., Cheng W. (2021). TiO_2_ nanotubes regulate histone acetylation through F-actin to induce the osteogenic differentiation of BMSCs. Artif. Cells Nanomed. Biotechnol..

[44] Kong K., Chang Y., Hu Y., Qiao H., Zhao C., Rong K., Zhang P., Zhang J., Zhai Z., Li H. (2022). TiO_2_ nanotubes promote osteogenic differentiation through regulation of Yap and Piezo1. Front. Bioeng. Biotechnol..

[45] Kunrath M.F., Vargas A.L.M., Sesterheim P., Teixeira E.R., Hubler R. (2020). Extension of hydrophilicity stability by reactive plasma treatment and wet storage on TiO_2_ nanotube surfaces for biomedical implant applications. J. R. Soc. Interface.

[46] Tang S., Wang Y., Zong Z., Ding N., Zhang Z. (2022). Enhanced osteogenic activity of titania-modified zirconia implant by ultraviolet irradiation. Front. Bioeng. Biotechnol..

[47] Lotz E.M., Olivares-Navarrete R., Berner S., Boyan B.D., Schwartz Z. (2016). Osteogenic response of human MSCs and osteoblasts to hydrophilic and hydrophobic nanostructured titanium implant surfaces. J. Biomed. Mater. Res. A.

[48] Parisi L., Ghezzi B., Bianchi M.G., Toffoli A., Rossi F., Bussolati O., Macaluso G.M. (2020). Titanium dental implants hydrophilicity promotes preferential serum fibronectin over albumin competitive adsorption modulating early cell response. Mater. Sci. Eng. C Mater. Biol. Appl..

[49] Wilson C.J., Clegg R.E., Leavesley D.I., Pearcy M.J. (2005). Mediation of biomaterial-cell interactions by adsorbed proteins: A review. Tissue Eng..

[50] Klein M.O., Bijelic A., Toyoshima T., Götz H., Von Koppenfels R.L., Al-Nawas B., Duschner H. (2010). Long-term response of osteogenic cells on micron and submicron-scale-structured hydrophilic titanium surfaces: Sequence of cell proliferation and cell differentiation. Clin. Oral Implants Res..

[51] Ryoo H.M., Lee M.H., Kim Y.J. (2006). Critical molecular switches involved in BMP-2-induced osteogenic differentiation of mesenchymal cells. Gene.

[52] Chen D., Gong Y., Xu L., Zhou M., Li J., Song J. (2019). Bidirectional regulation of osteogenic differentiation by the FOXO subfamily of Forkhead transcription factors in mammalian MSCs. Cell Prolif..

[53] Ching H.S., Luddin N., Rahman I.A., Ponnuraj K.T. (2017). Expression of odontogenic and osteogenic markers in DPSCs and SHED: A review. Curr. Stem Cell Res. Ther..

[54] Brogini S., Sartori M., Giavaresi G., Cremascoli P., Alemani F., Bellini D., Martini L., Maglio M., Pagani S., Fini M. (2021). Osseointegration of additive manufacturing Ti-6Al-4V and Co-Cr-Mo alloys, with and without surface functionalization with hydroxyapatite and type I collagen. J. Mech. Behav. Biomed. Mater..

[55] Li X., Xu H., Zhao B., Jiang S. (2018). Accelerated and enhanced osteointegration of MAO-treated implants: Histological and histomorphometric evaluation in a rabbit model. Int. J. Oral Sci..

[56] Thiem D.G., Adam M., Ganz C., Gerber T., Kämmerer P. (2019). The implant surface and its role in affecting the dynamic processes of bone remodeling by means of distance osteogenesis: A comparative in vivo study. Int. J. Oral Maxillofac. Implants.

[57] Takada S., Hirata E., Sakairi M., Miyako E., Takano Y., Ushijima N., Yudasaka M., Iijima S., Yokoyama A. (2021). Carbon nanohorn coating by electrodeposition accelerate bone formation on titanium implant. Artif. Cells Nanomed. Biotechnol..

[58] Khosravi N., DaCosta R.S., Davies J.E. (2021). New insights into spatio-temporal dynamics of mesenchymal progenitor cell ingress during peri-implant wound healing: Provided by intravital imaging. Biomaterials.

[59] Matsumoto T., Tashiro Y., Komasa S., Miyake A., Komasa Y., Okazaki J. (2020). Effects of surface modification on adsorption behavior of cell and protein on titanium surface by using quartz crystal microbalance system. Materials.

[60] Hyzy S.L., Cheng A., Cohen D.J., Yatzkaier G., Whitehead A.J., Clohessy R.M., Gittens R.A., Boyan B.D., Schwartz Z. (2016). Novel hydrophilic nanostructured microtexture on direct metal laser sintered Ti-6Al-4V surfaces enhances osteoblast response in vitro and osseointegration in a rabbit model. J. Biomed. Mater. Res. A.

